# Assessment of the Retromolar Canal in Taiwan Subpopulation: A Cross-Sectional Cone-Beam Computed Tomography Study in a Medical Center

**DOI:** 10.3390/tomography7020020

**Published:** 2021-05-31

**Authors:** Yen-Wen Shen, Wan-Chun Chang, Heng-Li Huang, Ming-Tzu Tsai, Lih-Jyh Fuh, Jui-Ting Hsu

**Affiliations:** 1 School of Dentistry, China Medical University, Taichung 404, Taiwan; a2312830@ms28.hinet.net (Y.-W.S.); u106000408@cmu.edu.tw (W.-C.C.); hlhuang@mail.cmu.edu.tw (H.-L.H.); 2 Department of Dentistry, China Medical University Hospital, Taichung 404, Taiwan; 3 Department of Bioinformatics and Medical Engineering, Asia University, Taichung 413, Taiwan; 4 Department of Biomedical Engineering, Hungkuang University, Taichung 433, Taiwan; anniemtt@sunrise.hk.edu.tw

**Keywords:** retromolar canal, dental cone-beam computed tomography, Taiwanese population

## Abstract

The retromolar canal is an anatomical variation that occurs in the mandibular bone. The retromolar canal typically originates in the mandibular canal on the distal side of the third molar and extends forward and upward to the retromolar foramen (RMF), which contains the neurovascular bundle. Accidentally damaging the neurovascular bundle in the retromolar canal during the extraction of the third molar, dental implant surgery, or maxillofacial orthognathic surgery may lead to subsequent complications such as incomplete local anesthesia, paresthesia, and bleeding during operation. The objective of this study was to investigate the prevalence of the RMF in the Taiwanese population in a medical center by using dental cone-beam computed tomography (CBCT) and to identify the position of the RMF in the mandibular bone. The dental CBCT images for the mandibular bone of 68 hemi-mandible were uploaded to the medical imaging software Mimics 15.1 to determine the prevalence of the RMF in the Taiwanese population and the three positional parameters of the RMF in the mandibular bone: (1) The diameter of the RMF, (2) the horizontal distance from the midpoint of the RMF to the distal cementoenamel junction of the second molar, and (3) the vertical distance from the midpoint of the RMF to the upper border of the mandibular canal. Seven RMFs were observed in the 68 hemi-mandibles. Thus, the RMF prevalence was 10.3%. In addition, the diameter of the RMF was 1.41 ± 0.30 mm (mean ± standard deviation), the horizontal distance from the midpoint of the RMF to the distal cementoenamel junction of the the second molar was 12.93 ± 2.87 mm, and the vertical distance from the midpoint of the RMF to the upper border of the mandibular canal below second molar was 13.62 ± 1.3487 mm. This study determined the prevalence of the RMF in the Taiwanese population in a medical center and its relative position in the mandibular bone. This information can provide clinicians with a reference for posterior mandible anesthesia and surgery to ensure medical safety.

## 1. Introduction

The retromolar fossa, in the form of a triangular depression, can be observed between the temporal crest medially and the anterior border of the ramus of the mandible laterally [1]. Clinically, this region is covered by elevated mucosa of variable size [2]. Clinicians should be aware of the regional anatomy and possible variations to avoid possible risks and complications in surgical procedures, such as impacted third molar extraction. The retromolar canal, an anatomical variation that occurs in the posterior mandible, is rarely mentioned in dentistry textbooks, and relevant studies have also highlighted numerous differences and uncertainties. The origin of the retromolar canal is explained by several hypotheses. First, Ossenberg et al. [3] discovered a high proportion of retromolar foramen (RMF) in adolescents’ mandible, and speculated that its appearance may be related to the sudden acceleration in physical development during adolescence and the adolescent growth spurt and eruption of the wisdom teeth that require more nerves and blood vessels. Ossenberg also argued that the appearance of the retromolar canal and the variability in RMF appear to be largely due to genetic differences. Chávez-Lomeli et al. [4] proposed that the mandibular canal is originally separated as at least three independent canals during the fetal period. At different stages of development, the three canals, each with their own independent foramens, connect to the incisors, primary molars, and permanent mandibular molars, respectively. When these canals are completely merged, a typical mandibular canal without any branches is formed. If these canals and their foramens do not undergo fusion and are retained, they may form a branched variation of the mandibular canal.

Several scholars have performed histopathologic investigations and verified that striated muscle fibers, myelinated nerve fibers, muscular arteries, and venules pass through the retromolar canal [5,6]. When a dentist is performing an operation, such as flap elevation with local anesthesia, osteotomy, insertion of dental implants, endodontic surgery, or surgical removal of roots or teeth and fails to notice the patient’s retromolar canal, injuring the anatomical structure may cause local anesthetic insufficiency or even hemorrhage during the operation [7]. Therefore, understanding the prevalence of the retromolar canal and its location is crucial [3,8,9,10,11,12].

The RMF is an opening at the retromolar canal located at the retromolar trigone behind the last mandibular molar [13,14,15,16]. Numerous approaches have been applied to determine the presence of RMF, such as the use of wires with a diameter of 0.5 mm to verify whether RMF is present on individuals’ dry mandible, panoramic radiograph, and measurement using cone-beam computed tomography (CBCT). The diverse research methods have resulted in a wide-ranging RMF prevalence rate (0–72%) [13]. Furthermore, because the retromolar canal and RMF are not present in all people, they are often overlooked. Relevant literature on the three-dimensional retromolar canal and two-dimensional RMF of the Asian population is rare. Thus, the objective of this study was to evaluate the prevalence of RMF in the Taiwanese population and the relative position of RMF, thereby providing a reference for dentists when performing anesthesia and treatments in the posterior mandible.

## 2. Materials and Methods

### 2.1. Dental CBCT Examinations

Dental CBCT images were obtained from 68 Taiwanese patients (30 men and 38 women) at China Medical University Hospital, Taiwan. The CBCT scans were performed using the Asahi AZ3000 (Asahi Roentgen Ind. Co., Kyoto, Japan) with the following technical parameters: 85 kV, 12.5 mA, 155 µm voxel resolution, and 70 mm field of view (FOV). All patients underwent CBCT scans as part of surgical assessments for dental implant insertion or endodontic treatment. Because of the small FOV, each dental CBCT only provided an image of one side of the mandible. Therefore, each dental CBCT only imaged the left or right mandible. Thus, 68 hemi-mandibles were evaluated in this study. Of the 68 CBCT images, 33 images (of 13 men and 20 women) and 35 images (of 17 men and 18 women) were of the left and right side of the mandible, respectively. This study was executed with the ethical approval of the Institutional Review Board of China Medical University Hospital (CMUH108-REC2-083).

### 2.2. Measurement of the Prevalence of the Retromolar Canal and its Spatial Position in the Mandible

**Figure 1:**
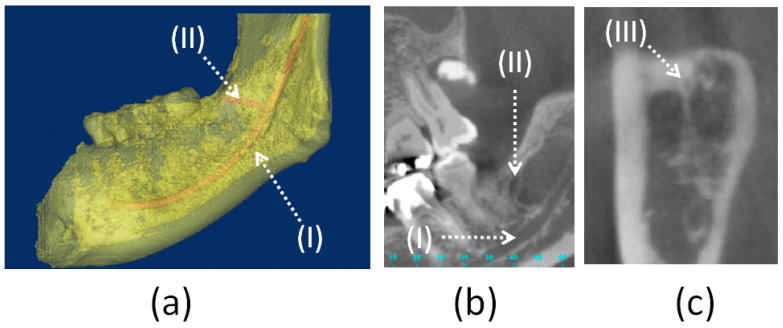
Mandibular canal (I), retromolar canal (II), and retromolar foramen (III) in the right side of the mandibular bone: (**a**) Three-dimensional model, (**b**) tangential plane, (**c**) coronal plane.

**Figure 2:**
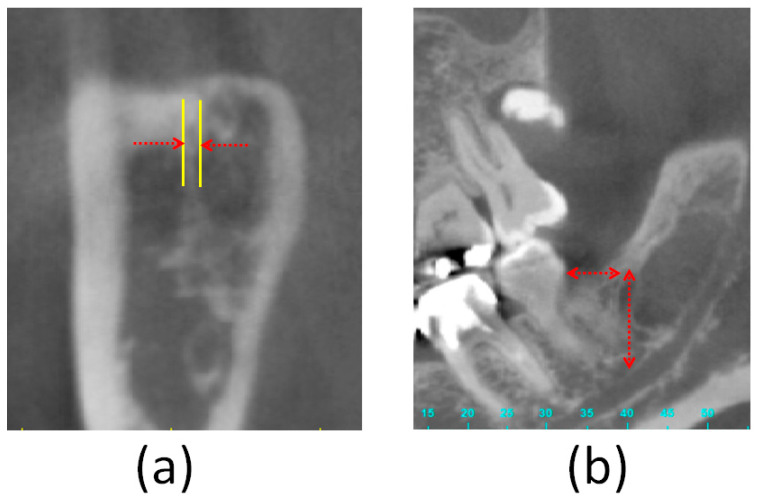
Measurement of the three parameters of the retromolar foramen: (**a**) The diameter of the retromolar foramen, (**b**) the horizontal distance from the midpoint of the retromolar foramen to the distal cementoenamel junction of the second molar, (I) and the vertical distance from the midpoint of the retromolar foramen to the upper border of the mandibular canal below the second molar (II).

The dental CBCT images were uploaded to the medical imaging software Mimics 15.0 (Materialise, Leuven, Belgium) to calculate the prevalence rate of the RMF in the 68 patients. Seven of the 68 hemi-mandibles exhibited RMF. In these seven hemi-mandibles, the following three parameters were measured: (1) the diameter of the RMF, (2) the horizontal distance from the midpoint of the RMF to the distal cementoenamel junction (CEJ) of the second molar, and (3) the vertical distance from the midpoint of the RMF to the upper border of the mandibular canal below the second molar (Figure 1 and Figure 2).

### 2.3. Statistical Analysis

The Shapiro–Wilk test was used to verify that all measurements conformed to a normal distribution. Therefore, the mean and standard deviation were calculated for all measurements. For the three parameters, the two-sample t-test was used to compare the differences between the left and right sides of the mandible and between men and women. All statistical analyses were conducted using SPSS version 19 (IBM Corporation, Armonk, NY, USA), and the significance level was set to *p* < 0.05.

## 3. Results

In terms of the prevalence of RMFs in different sexes, three and four RMFs were observed in the hemi-mandibles of 30 men and 38 women, respectively, indicating a respective RMF prevalence of 10% and 10.5%. This finding indicated that sex had no influence on RMF prevalence. Moreover, in terms of RMF prevalence in the left and right mandibles, three RMFs were observed in 33 left mandibles (9.1%), and four RMS were recorded in 35 right mandibles (11.4%). Therefore, the side of the mandible had no effect on RMF prevalence.

RMFs were observed in seven of the 68 hemi-mandibles. Table 1 presents the three parameters related to the RMF of the seven patients, namely the diameter of the RMF, the horizontal distance from the midpoint of the RMF to the distal cementoenamel Junction (CEJ) of the second molar, and the vertical distance from the midpoint of the RMF to the upper border of the mandibular canal below the second molar, which were 1.41 ± 0.30 mm, 11.57 ± 2.70 mm, and 13.62 ± 1.34 mm, respectively. In addition, in the seven patients with RMF, no significant difference was observed between men and women for the aforementioned parameters (diameter of the RMF, *p* = 0.43; horizontal distance from the midpoint of the RMF to the distal CEJ of the second molar, *p* = 0.38; and vertical distance from the midpoint of the RMF to the upper border of the mandibular canal below the second molar, *p* = 0.91). In terms of the left and right sides of the mandibular, except for the RMF diameter (*p* = 0.03), the horizontal distance from the midpoint of the RMF to the distal CEJ of the second molar (*p* = 0.58) and the vertical distance from the midpoint of the RMF to the upper border f the mandibular canal below the second molar (*p* = 0.12) did not exhibit any significant difference.

## 4. Discussion

The retromolar canal typically originates in the mandibular canal of the distal second or third mandibular molar, and extends upward and forward to the RMF, which contains the neurovascular bundle. Injuring the neurovascular bundle in the retromolar canal during the extraction of an impacted third molar, dental implant surgery, or maxillofacial orthognathic surgery may result in local anesthetic insufficiency, paresthesia, and more bleeding during operation. Thus, devising a posterior mandibular anesthesia and treatment plan is a crucial reference factor. However, relatively few studies have explored this issue because of the rarity of the mandibular canal. This study is the first to use CBCT to investigate the prevalence of the retromolar canal in Taiwanese people and the position of the retromolar canal in the mandible. The experimental results indicated a 10.3% prevalence for the retromolar canal in the Taiwanese population. Furthermore, sex and the left and right sides of the mandible had no significant effect on the incidence of the retromolar canal. Interestingly, there was no patient with RMF in both right and left mandible. The findings of this study could serve as a reference for Taiwanese dentists when performing posterior mandible anesthesia and surgery, as well as provide reference data for future interracial or intergroup cross-comparison research.

Clinically, if a dentist fails to notice the patient’s retromolar canal and injures its neurovascular bundle during surgery, postoperative paresthesia or excessive bleeding may occur [5]. Von Arx et al. [13] noted that a greater understanding of the retromolar canal may help practitioners recognize problems such as incomplete anesthesia in the local area and bleeding. They [13] also advised clinicians to preserve this anatomic variation when performing surgery in the retromolar area and to consider additional local anesthesia in case of failed mandibular block anesthesia. If anesthesia insufficiency occurs for mandibular block, the dentist should consider the possibility of the existence of a retromolar canal. Scholars have attempted to investigate the RMF, an opening at the retromolar canal. However, their studies have yielded a wide-ranging RMF prevalence (1.7–72%), which was likely caused by the different research methods adopted. Von Arx et al. [13] employed CBCT and panoramic radiography to calculate the prevalence of the retromolar canal. RMF was observed in 25.6% and 5.8% of patients when using CBCT and panoramic radiography, respectively. Han et al. [17] also demonstrated that CBCT images are more effective than panoramic radiographs for evaluating the prevalence of retromolar canals. Haas et al. [18] indicated two types of variation in the mandibular canal: the retromolar canal and bifid mandibular canal. They concluded that CBCT images are considerably more accurate than panoramic radiography for detecting these small canals. Fukami et al. [6] also remarked that the retromolar canal was more easily observed with CBCT than with clinical medical computed tomography (CT). This could be attributed to the resolution of dental CBCT being higher than that of CT. Accordingly, this study adopted CBCT as the research method to identify the presence of the RMF in the Taiwanese population.

In terms of the relationship between sex and the incidence of RMF, despite the research by Orhan et al. [19] indicating that the proportion of women with RMF (19%) was slightly higher than that of men (15.4%), most scholars (e.g., Ossenberg et al. [3], von Arx et al. [13], Pyle et al. [20], Kodera et al. [21]) have concluded nonsignificant differences between different sexes in the incidence of RMF. In this study, the prevalence of RMF in Taiwanese men and women was 10.0% and 10.5%, respectively, which was similar to the results of most studies, and no statistically significant difference was observed.

In investigating the relationship between the left and right mandible and the incidence of RMF, Ossenberg [3] studied 2500 mandibular bones and discovered that in groups with a low RMF prevalence rate, the proportion of RMF in the right mandible was higher, whereas the groups with a high RMF prevalence rate was found to have a high proportion of RMF in their left mandibles (Table 2). Narayana et al. [22] and Orhan et al. [19] concluded that the proportion of RMF found in the left mandible was higher than that in the right mandible (Table 2). However, Park et al. [23] and von Arx et al. [13] discovered that the prevalence rate of the RMF on both sides of the mandible was not significantly different. This is similar to the current findings, wherein the prevalence rate of the RMF in the right mandible (11.4%) was higher than that in the left mandible (9.1%). However, the difference was not significant (Table 2). No participant in this study exhibited the RMF in both the left and right sides of the mandible. Even though the current finding differs from that of Ossenberg, the two studies have compatible conclusions: RMFs occurred more often unilaterally than bilaterally.

In terms of the relationship between different race and region and the prevalence of RMF (Table 3), Ossenberg (1987) suggested that the prevalence of the RMF in native North Americans was higher than that in people from other regions, such as Africa, Europe, India, and Northeast Asia. Notably, both Ossenberg [3] and Kodera and Hashimoto [21] studied the Japanese population but reached different conclusions on the prevalence of RMF in that group (5.4% and 19.5%, respectively). This disparity could be attributed to the different methods adopted in the two studies in their evaluation of the RMF. In the present study, CBCT revealed that seven out of the 68 participants had an RMF. That is, the prevalence of the RMF in Taiwanese people is 10.3%, which is similar ti that in Korean people (11.5%), who are also Asian.

In a study on the relative position of the RMF in the mandible, von Arx et al. [13] measured 121 samples by using CBCT images; the results indicated that age affected the horizontal distance from the RMF to the second molar. Specifically, the researchers discovered that compared with older patients, the horizontal distance from the RMF to the second molar was longer in younger patients, which was possibly because the wisdom teeth of the younger patients had not been extracted yet. The second molar may undergo slight migration or distal tipping after a wisdom tooth is extracted, leading to a shortened horizontal distance from the RMF to the second molar [3]. Among the seven patients (out of 68 patients) who had an RMF, the diameter of the RMF was 1.41 ± 0.30 mm, the horizontal distance from the midpoint of the RMF to the distal CEJ of the second molar was 11.57 ± 2.70 mm, and the vertical distance from the midpoint of the RMF to the upper border of the mandibular canal was 13.62 ± 1.3487 mm. These values differed from those obtained by von Arx et al. (2011), who similarly used CBCT (0.99 ± 0.31 mm, 15.16 ± 2.39 mm, and 11.34 ± 2.36 mm, respectively). The current authors speculate that this difference is related to the race of the measured sample.

In terms of the clinical implications of this study, dentists performing surgical procedures, including third molar extraction, harvesting of autologous bone from the retromolar area for grafts, sagittal splitting osteotomy [5,24,25], and removable denture fabrication [8], should be aware of this anatomical landmark. This knowledge can help prevent incomplete mandibular block anesthesia and injury to this area’s neurovascular bundle leading to hemorrhages, dysesthesias, and even removable denture base or flange impingement.

This study has some limitations. First, the participants in this study were Taiwanese of Mongolian descent. Thus, the current findings may not be applicable to other races. Second, because of the small sample size, this study did not include a statistical comparison by age. Future studies may increase the sample size to 500–1000 and also divide the participants into subgroups according to age.

## 5. Conclusions

The results of this study revealed that the prevalence of the RMF in Taiwanese people is 10.3% and that sex, as well as the left and right sides of the mandible, do not have a significant influence on the prevalence of RMF. The diameter of the RMF was 1.41 ± 0.30 mm, the horizontal distance from the midpoint of the RMF to the distal CEJ of the second molar was 11.57 ± 2.70 mm, and the vertical distance from the midpoint of the RMF to the upper border of the mandibular canal below the second molar was 13.62 ± 1.3487 mm. The current findings serve as a reminder for Taiwanese dentists to be cautious when administering mandibular anesthesia and performing surgeries in the posterior area of the mandible to ensure patient safety.

## Figures and Tables

**Table 1:** The diameter of the retromolar foramen and its spatial position in the mandibular bone.

Number	Age	Which Side of Mandible	Diameter of Retromolar Foramen (mm)	Horizontal Distance from Retromolar Canal to Second Molar (mm)	Height of Retromolar Canal (mm)
Case 1	50~55	Left	1.56	13.89	12.86
Case 2	45~50	Right	1.49	9.02	15.43
Case 3	45~50	Right	1.14	Missing tooth	14.3
Case 4	55~60	Left	1.57	8.01	12.83
Case 5	20~25	Left	1.86	11.02	12.43
Case 6	20~25	Right	1.01	11.58	15.25
Case 7	40~45	Right	1.26	15.92	12.27
			1.41 ± 0.30	11.57 ± 2.70	13.62 ± 1.34

**Table 2:** Comparison of the prevalence rates of the retromolar foramen and retromolar canal in the right and left mandible between related literature and this study.

Researchers	Method (Sample Size)	Right Side (%)		Left Side (%)
Ossenberg et al. [3]	Dry mandible (2500)	Old world	60.0	25.7
			Bilateral: 14.3	
		New World	25.9	40.5
			Bilateral: 33.5	
Suazo et al. [12]	Dry mandible (294)	4.8		4.4
		Bilateral: 3.7		
von Arx et al. [13]	CBCT (121)	23.3		27.9
Orhan et al. [19]	CBCT (484)	20.4		17.7
Park et al. [23]	Dry mandible (140)	32.9		34.3
Narayana et al. [22]	Dry mandible (242)	10.7		7.1
		Bilateral: 4.1		
This study	CBCT (68)	11.4		9.1

**Table 3:** Prevalence of the retromolar foramen in the mandible in different populations from the literature and this study.

Researcher	Measurement Approach	Population		Sample Size *	Percentage (%)
Ossenberg et al. [3]	Dry mandible	Black Africans		19 *	0
		Black Americans		33 *	0
		North American native peoples, prehistoric and present	North American native peoples	99 *	1.0
			Plain Indians	435 *	8.0
			Northern Indians	178 *	15.2
			Eskimo	485 *	8.2
			Aleut	192 *	15.1
		Canadian dissecting-room subjects		11 *	9.1
		India		153 *	5.9
		Italians, Siena		86 *	8.1
		Siberian native people		167 *	3.6
		Japanese		94 *	3.2
Pyle et al. [20]	Dry mandible	Caucasian and African American		475 *	7.8
Suazo et al. [12]	Dry mandible	Brazilians		294 *	12.9
von Arx et al. [13]	CBCT	Not available		121 (sides)	25.6
Orhan et al. [19]	CBCT	Turkish		242 *	66.5
				484 (sides)	46.5
Park et al. [23]	CBCT	Korean		100 *	11.5
	Micro-computed tomography	Korean		22 (sides)	68.1
	Dry mandible	Korean		140 (sides)	33.6
Narayana et al. [22]	Dry mandible	South Indian		242 *	21.9
This study	CBCT	Taiwanese		68 (sides)	10.3

* Note: number of mandibles.

## Data Availability

The data used to support the findings of this study are available from the corresponding author upon request.
